# Ado-trastuzumab for the treatment of metastatic HER2-positive breast cancer in patients previously treated with Pertuzumab

**DOI:** 10.1186/s12885-021-08894-2

**Published:** 2021-10-27

**Authors:** Luai S. Al Rabadi, Madeline M. Cook, Andy J. Kaempf, Megan M. Saraceni, Michael A. Savin, Zahi I. Mitri

**Affiliations:** 1 Knight Cancer Institute, Oregon Health & Science University, 3181 SW Sam Jackson Road, OC14HO, Portland, OR 97239 USA; 2 School of Medicine, Oregon Health & Science University, Portland, OR USA; 3 Knight Cancer Institute, Biostatistics Shared Resource, Oregon Health & Science University, Portland, OR USA; 4 Department of Pharmacy, Oregon Health & Science University, Portland, OR USA

**Keywords:** Breast Cancer, HER2, T-DM1, Ado-Trastuzumab, Pertuzumab

## Abstract

**Background:**

Docetaxel in combination with two HER2-directed therapies, trastuzumab and pertuzumab, is the current standard frontline therapy for patients with metastatic HER2-positive breast cancer. Ado-trastuzumab (T-DM1), an antibody-drug conjugate of trastuzumab and a cytotoxic microtubule-inhibitory agent, emtansine, is approved in patients that have progressed with prior trastuzumab-based therapy. However, the benefit of T-DM1 in patients previously treated with pertuzumab therapy for metastatic breast cancer remains unclear.

**Methods:**

We identified thirty-three adults with metastatic HER2-positive breast cancer treated between March 2013 and July 2018 with T-DM1 either as subsequent therapy after progression on a pertuzumab-based regimen (i.e., “pertuzumab-pretreated”) or without prior exposure to pertuzumab (i.e., “pertuzumab-naïve”). Collected data included patient demographics, treatment history, adverse events, and clinical outcomes. For both cohorts receiving T-DM1, the primary endpoint was PFS and secondary endpoints were overall survival (OS), overall response rate (ORR), clinical benefit rate (CBR), and T-DM1-related toxicity rate.

**Results:**

Pertuzumab-pretreated patients (*n* = 23, with 21 evaluable for T-DM1 efficacy) had a median PFS of 9.5 months (95% CI: 2.9–NA), 1-year OS rate of 67.4% (95% CI: 50.0–90.9%) with an unreached median, ORR of 14.3% (95% CI: 3.0–36.3%), and CBR of 52.4% (95% CI: 29.8–74.3%), with none of these measures being statistically different than those estimated for the pertuzumab-naïve group (*n* = 10). Treatment with T-DM1 after prior pertuzumab exposure (median T-DM1 duration 2.9 months) resulted in no grade ≥ 3 adverse events.

**Conclusions:**

In our cohort, prior exposure to pertuzumab did not significantly impact T-DM1’s clinical efficacy or safety profile as second- or later-line therapy in patients with metastatic HER2-positive breast cancer.

## Background

The gene that encodes human epidermal growth factor receptor 2 (HER2) is amplified or overexpressed in approximately 15–20% of invasive breast cancer cases and historically associated with an increased risk of disease recurrence and overall worse prognosis than HER2-negative breast cancer [1, 2]. The development of HER2-directed therapies has altered the natural history of HER2-positive breast cancer and has led to a continued improvement in outcomes for this disease historically associated with a poor prognosis [3–6].

The current standard first-line therapy for patients with metastatic HER2-positive breast cancer consists of the chemotherapy agent docetaxel in combination with two HER2-directed therapies, trastuzumab and pertuzumab, and is continued until disease progression or unacceptable toxicity. This standard is based on results of the CLEOPATRA trial, a phase III randomized study which demonstrated that the addition of pertuzumab to trastuzumab and docetaxel conferred both a progression-free survival (PFS) and overall survival (OS) benefit compared to trastuzumab and docetaxel alone [7, 8].

Ado-trastuzumab (T-DM1), an antibody-drug conjugate of trastuzumab and a cytotoxic microtubule-inhibitory agent, emtansine (DM1), has been shown to prolong PFS when studied in second-line (EMILIA) and later-lines of therapy (TH3RESA) in metastatic HER2-positive breast cancer [9, 10]. However, these trials were conducted prior to the widespread adoption of docetaxel, trastuzumab and pertuzumab as frontline therapy. Therefore, the clinical benefit of T-DM1 in patients previously treated with pertuzumab therapy for metastatic breast cancer remains uncertain. Herein this article, we review our institution’s experience of patients treated with T-DM1 with and without prior treatment with pertuzumab for metastatic HER2-positive breast cancer.

## Methods

### Patient population

Between March 2013 and July 2018, electronic pharmacy records, electronic medical records, and departmental databases from Oregon Health and Science University (OHSU) were reviewed to identify patients at least 18 years of age with metastatic HER2-positive breast cancer who received T-DM1 during that period either: (i) as subsequent therapy after progression on a pertuzumab-based regimen (i.e., “pertuzumab-pretreated”) or (ii) without prior exposure to pertuzumab (i.e., “pertuzumab-naïve”). The cutoff date for collection of patient follow-up data was July 1st, 2019. OHSU’s institutional review board’s approval and waiver of informed consent were obtained prior to commencing the chart review.

### Data collection

Extracted patient data from both chart notes and medication administration records within the electronic medical record was collected and entered into a database containing the following fields: patient demographics, tumor characteristics (hormone receptor status, HER2 status by immunohistochemistry (IHC) and/or fluorescence in situ hybridization (FISH) if assessed using 2010 and 2013 ASCO/College of American Pathologists guidelines, respectively), site and date of metastatic recurrence, treatment history (i.e. prior chemotherapy, anti-HER2 therapy, endocrine therapy, lines of treatment for metastatic breast cancer), adverse events of special interest while on T-DM1 therapy (using CTCAE 4.03), dose reductions, and reason for treatment discontinuation. Disease response was determined by the healthcare provider in the context of routine care and imaging reports collected during T-DM1 therapy to measure tumor response based on RECIST criteria (version 1.1). Dates of last contact, disease progression, last exam, and death (if applicable) were collected from the electronic medical records, until the July 1st, 2019 cutoff date.

### Statistical analysis

For both cohorts of patients receiving T-DM1, the primary endpoint was PFS and secondary endpoints were OS, overall response rate (ORR), clinical benefit rate (CBR), and T-DM1-related toxicity rate. The date of initial T-DM1 infusion was defined as the start time for PFS and OS analyses. Patient demographic, tumor, and treatment characteristics were summarized by cohort and compared across cohorts using Fisher’s exact test (for categorical variables) and the Kruskal-Wallis test (for continuous variables). Confidence intervals for proportions of patients achieving a binary outcome were estimated using the Clopper-Pearson method.

Progression-free survival was calculated as the elapsed time between T-DM1 initiation and radiographically-confirmed disease progression, death without progression, or last clinical exam (for those patients without a documented progression or death). Time-to-event outcomes (i.e., PFS and OS) were estimated and plotted according to the Kaplan-Meier method, compared with various Fleming-Harrington non-parametric tests such as the log-rank (for categorical predictors), and modeled by Cox proportional hazards regression. Associations between baseline (i.e., known at the time of T-DM1 initiation) patient characteristics and survival outcomes were assessed with hazard ratios (HR’s) from Cox models upon checking the proportional hazards assumption by visual inspection and testing of variance-scaled Schoenfeld residuals from uncensored patients. Effects corresponding to *p*-values < 0.05 were deemed statistically significant and no multiplicity adjustment of *p*-values was performed.

## Results

### Pertuzumab-pretreated cohort

The cohort that received T-DM1 after progressing on a pertuzumab-containing regimen consisted of 23 women with a median age of 58 years at the time of metastatic diagnosis (range 34 to 86 years). The median time from initial breast cancer diagnosis to detection of metastases was two years (range 0 to 16 years), including 10 women diagnosed with de novo metastatic disease. The number of pre-T-DM1 systemic therapies (excluding pertuzumab) in the metastatic setting ranged from 0 (*n* = 6) to 8 with a median of 1. At the time of T-DM1 initiation, common sites of metastases in this cohort were the lungs (69.6% of women), bones (65.2%), liver (47.8%), and brain (43.5%). Patient-level characteristics are further summarized by treatment cohort in Table 1.

**Table 1:** Descriptive statistics

Characteristic	Pertuzumab-pretreated (*n* = 23)	Pertuzumab-naive (*n* = 10)	*P* value*
Months from initial	median: 23.5	median: 14.7	0.763
Dx to Met Dx	range: 0.0–192.5	range: 0.0–53.6	
Initial Dx of Met	No: 13 (56.5%)	No: 8 (80.0%)	0.259
disease	Yes: 10 (43.5%)	Yes: 2 (20.0%)	
Age at Met Dx	median: 58.0	median: 52.0	0.377
	range: 34.0–86.0	range: 38.0–67.0	
Ethnicity	white: 20 (87.0%)	white: 9 (90.0%)	1.000
	black: 3 (13.0%)	black: 1 (10.0%)	
Num. prior therapies	median: 1.0	median: 1.0	0.586
in Met setting	range: 0.0–8.0	range: 0.0–7.0	
Num. prior therapies	0: 6 (26.1%)	0: 1 (10.0%)	0.397
in Met setting (binary)	> = 1: 17 (73.9%)	> = 1: 9 (90.0%)	
Brain mets	No: 13 (56.5%)	No: 6 (60.0%)	1.000
(at T-DM1 start)	Yes: 10 (43.5%)	Yes: 4 (40.0%)	
Bone mets	No: 8 (34.8%)	No: 6 (60.0%)	0.257
(at T-DM1 start)	Yes: 15 (65.2%)	Yes: 4 (40.0%)	
Lung mets	No: 7 (30.4%)	No: 9 (90.0%)	0.002
(at T-DM1 start)	Yes: 16 (69.6%)	Yes: 1 (10.0%)	
Liver mets	No: 12 (52.2%)	No: 6 (60.0%)	0.722
(at T-DM1 start)	Yes: 11 (47.8%)	Yes: 4 (40.0%)	
HR+ (ER+ or PR+)	No: 12 (52.2%)	No: 2 (25.0%)	0.240
at metastasis	Yes: 11 (47.8%)	Yes: 6 (75.0%)	
Months from Met Dx	median: 12.6	median: 8.2	0.845
to T-DM1 therapy	range: 0.3–69.8	range: 0.4–60.2	
Months from Met Dx	< 10: 10 (43.5%)	< 10: 6 (60.0%)	0.465
to T-DM1 (binary)	> = 10: 13 (56.5%)	> = 10: 4 (40.0%)	
Months of T-DM1	median: 2.9	median: 4.8	0.799
therapy	range: 0.7–50.4	range: 0.7–40.8	
T-DM1 dose	No: 18 (78.3%)	No: 9 (90.0%)	0.640
reduction	Yes: 5 (21.7%)	Yes: 1 (10.0%)	
Cardiac toxicity	No: 22 (95.7%)	No: 8 (80.0%)	0.212
(during T-DM1)	Yes: 1 (4.3%)	Yes: 2 (20.0%)	
Neuropathy	No: 21 (91.3%)	No: 9 (90.0%)	1.000
(during T-DM1)	Yes: 2 (8.7%)	Yes: 1 (10.0%)	
T-DM1 discontinuation	No: 22 (95.7%)	No: 9 (90.0%)	0.521
due to toxicity	Yes: 1 (4.3%)	Yes: 1 (10.0%)	
Overall response	No: 18 (85.7%)	No: 7 (70.0%)	0.358
(CR or PR)	Yes: 3 (14.3%)	Yes: 3 (30.0%)	
CBR (CR, PR, or SD	No: 10 (47.6%)	No: 5 (50.0%)	1.000
with T-DM1 > 6 mo)	Yes: 11 (52.4%)	Yes: 5 (50.0%)	

* *P* values from Fisher’s exact test (for categorical variables) or Kruskal-Wallis test (for continuous variables)

The median time between diagnosis of metastatic disease and the start of T-DM1 in this pertuzumab-pretreated group was 12.6 months (range < 1 to 70 months). The duration of T-DM1 therapy varied from 3 weeks to 4 years with a median of 2.9 months. Ten of the 23 women (43%) were given T-DM1 for more than 6 months. Patient follow-up (starting from the first T-DM1 infusion) ranged from 1 to 50 months (median 17). T-DM1-related adverse events included one patient with grade 1 cardiac dysfunction and two patients with grade ≤ 2 peripheral sensory neuropathy. Five patients (21.7%) required a T-DM1 dose reduction. Among the 21 women formally evaluated for response to T-DM1 (RECIST v1.1), there were no complete responses, 3 patients had a partial response, and 8 other patients had stable disease with > 6 months of T-DM1 treatment, leading to an overall response rate of 14.3% (95% CI: 3.0–36.3%) and clinical benefit rate of 52.4% (95% CI: 29.8–74.3%).

### Pertuzumab-Naïve cohort (control)

The concurrent control group of HER2-positive breast cancer patients administered T-DM1 without antecedent pertuzumab in the metastatic setting consisted of 10 women, 2 of whom were diagnosed with de novo metastatic disease. With a median age of 52 years (range 38 to 67), this cohort was younger yet not statistically different than the pertuzumab-pretreated group. Pertuzumab-naive women received from 0 (*n* = 1) to 7 systemic therapies (median 1) between metastatic diagnosis and commencement of T-DM1.

Median duration of T-DM1 therapy was 4.8 months (range 3 weeks to 41 months). As in the pertuzumab group, T-DM1 was well tolerated, with only one out of ten patients requiring T-DM1 dose reduction. T-DM1 related adverse events included two patients experiencing cardiac dysfunction (both grade 1) and one with peripheral sensory neuropathy (grade 2). The overall response rate was 30.0% (95% CI: 6.7–65.2%) and clinical benefit rate was 50.0% (95% CI: 18.7–81.3%) based on three pertuzumab-naïve patients achieving a partial response and two others having stable disease while receiving T-DM1 for greater than 6 months.

The only patient characteristic that significantly differed between cohorts was metastasis to the lungs (*p* = 0.002, Table 1), which was observed in 10% of patients in the control group compared to 70% of pertuzumab-pretreated patients. All other patient features were similar across the two patient groups (*p*-values > 0.200, Table 1).

### Survival outcomes

**Fig. 1:**
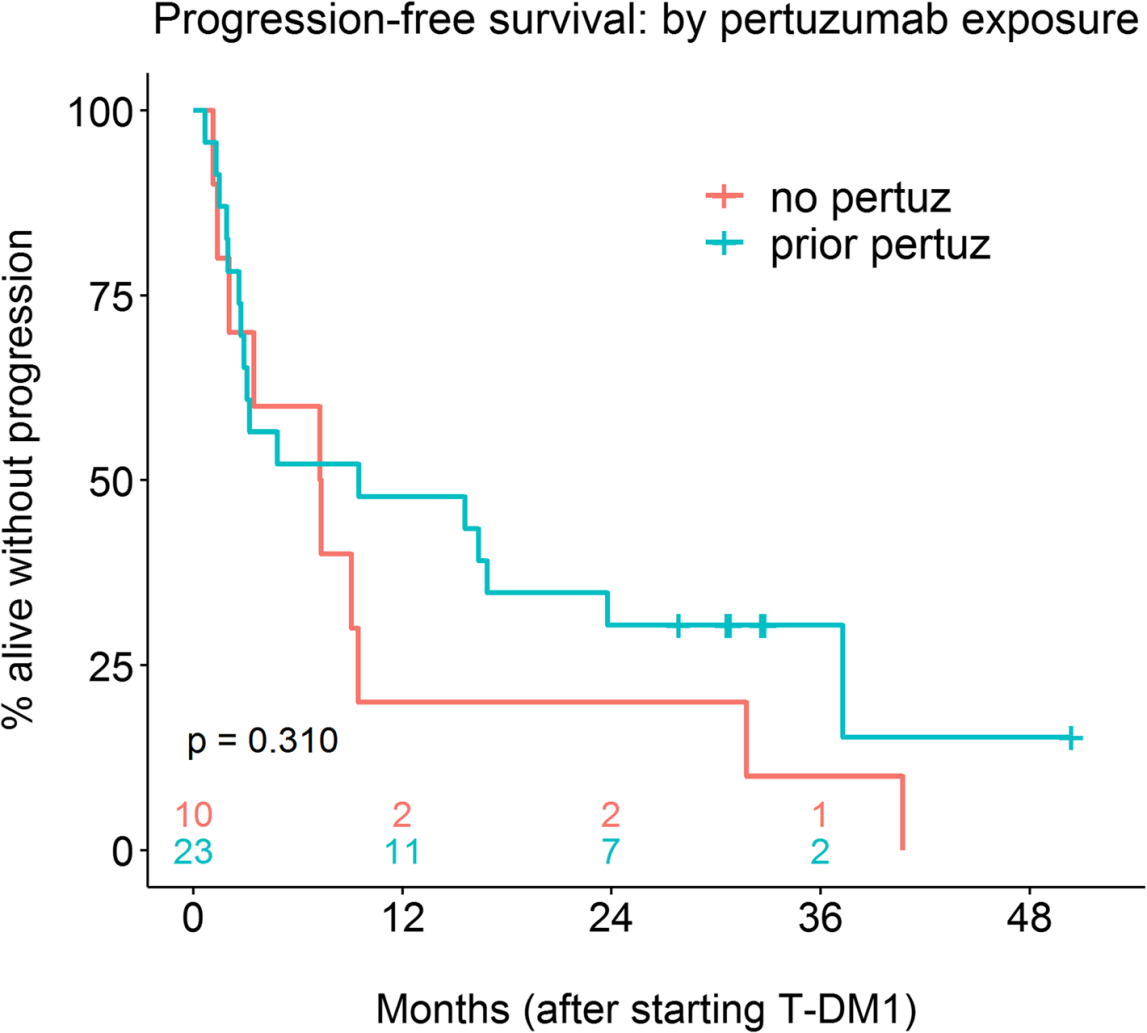
Progression free survival for pertuzumab pre-treated and pertuzumab-naive patients

Among the 23 pertuzumab-pretreated patients, the one-year PFS rate was 47.8% (95% CI: 31.2–73.3%) with a median PFS of 9.5 months (Fig. 1). Within this cohort, black race (HR = 4.02 [95% CI: 1.07–15.10] compared to white; *p* = 0.026) and liver metastasis (HR = 7.78 [95% CI: 2.07–29.26]; *p* < 0.001) were significantly associated with worse PFS (Table 2). Among the 10 pertuzumab-naïve patients, the 1-year PFS rate was 20.0% (95% CI: 5.8–69.1%), with a median PFS of 7.3 months. Thus, the pertuzumab-pretreated group had a favorable, albeit non-significant, PFS distribution compared to the pertuzumab-naïve group (HR = 0.66 [95% CI: 0.30–1.47]; *p* = 0.310; Table 2). Starting at 9 months after T-DM1 initiation, the proportion of patients who were alive and progression-free was greater in the pertuzumab-pretreated cohort (Fig. 1). However, 11 of the 17 PFS events in the pertuzumab group occurred before 9 months and the Prentice modification test that assigns more weight to earlier differences between groups had a *p*-value of 0.500 (> log-rank p-value of 0.310). Interestingly, there was a strong interaction effect on PFS between pertuzumab exposure and hepatic malignancy; pertuzumab-naïve patients with liver metastasis at the start of T-DM1 had a reduced risk of disease progression or death (HR = 0.20 [95% CI: 0.04–0.88]; *p* = 0.033; Table 2) compared to other control group women, which was contrary to the above mentioned greater risk of progression or death for pertuzumab-pretreated women with cancer in the liver.

**Table 2:** Progression free survival

Covariate(s) in model	Patients	Hazard Ratio (Pertuz vs. Control)	HR 95% CI	*P* value
None	All	0.66	0.30–1.47	0.310
Months from initial Dx to Met Dx	All	0.78	0.35–1.76	0.549
Race	All	0.59	0.25–1.38	0.222
Liver Met; interaction	Liver mets	4.26	1.10–16.60	0.036
Liver Met; interaction	No liver mets	0.11	0.03–0.42	0.001

**Fig. 2:**
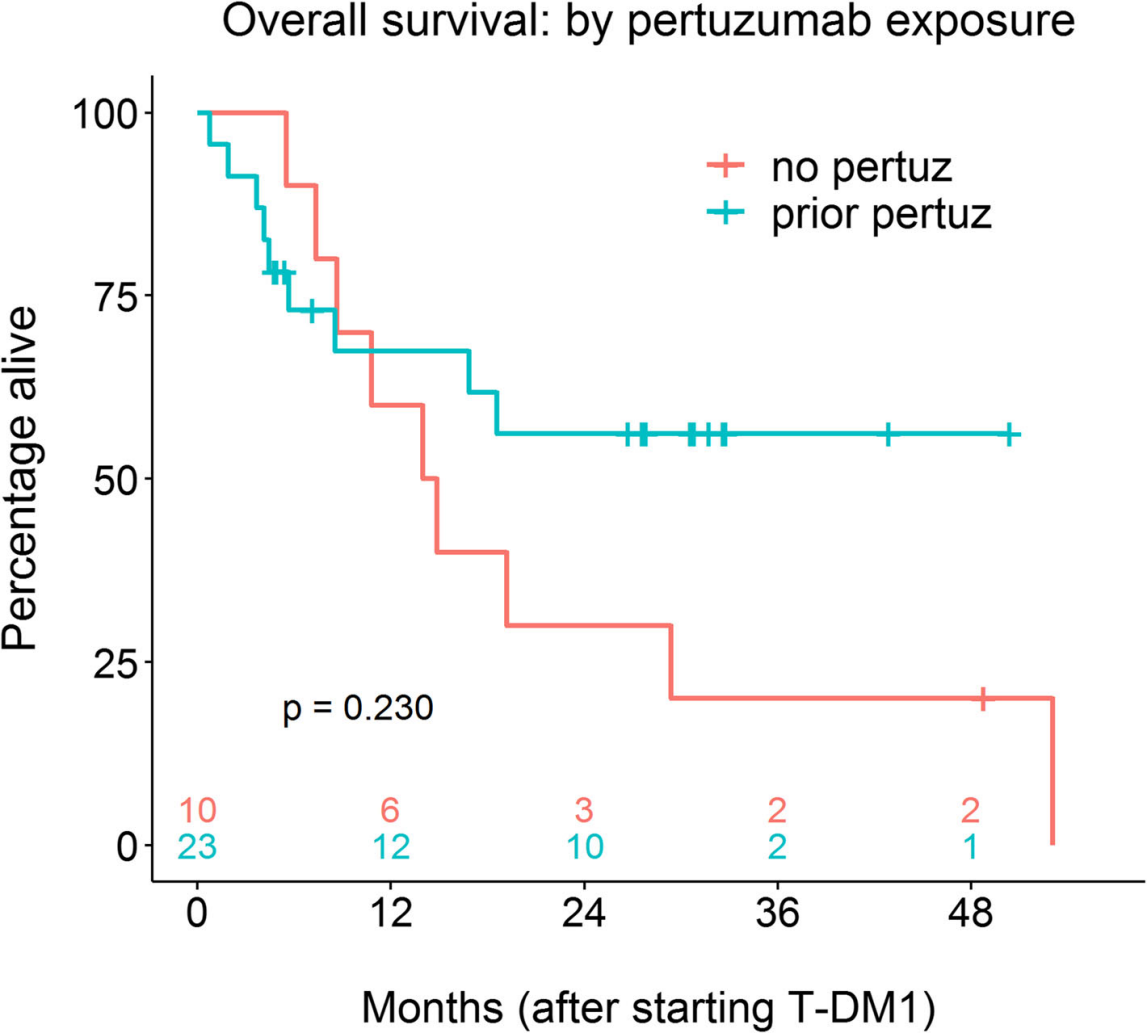
Overall survival for pertuzumab pre-treated and pertuzumab-naive patients

There were 9 observed deaths among the 23 women in the pertuzumab-pretreated group, with deaths occurring 0.8 to 18.6 months after start of T-DM1 therapy (median 4.4 months). Median follow-up for this group was 16.9 months. The one-year OS rate was 67.4% (95% CI: 50.0–90.9%) and median OS was not reached (Fig. 2). None of the baseline patient demographic or disease features were associated with OS in the pertuzumab-pretreated cohort. Among the 10 pertuzumab-naïve patients, there were 9 deaths (range 5.5 to 53.1 months after starting T-DM1; median 14.0 months) and both median follow-up and median OS were 14.4 months. Patients with exposure to pertuzumab had higher 1-year (67.4% vs 60.0%) and 2-year (56.2% vs 30.0%) OS rates compared to the pertuzumab-naïve group; however, when evaluated over the entire follow-up period, this survival advantage was not statistically significant (HR = 0.56 [95% CI: 0.22–1.46]; *p* = 0.230; Table 3).

**Table 3:** Overall survival

Covariate in model	Patients	HR (Pertuz vs. Control)	HR 95% CI	P value
None	All	0.56	0.22–1.46	0.230
Number prior therapies in Met setting	All	0.57	0.22–1.50	0.257

Compared to the respective univariable model, there was minimal change in the association between pertuzumab exposure and each time-to-event outcome when adjusting for lung metastasis (the only patient feature that significantly differed across treatment cohorts) as a covariate: PFS HR = 0.46 (95% CI: 0.17–1.20), OS HR = 0.47 (95% CI: 0.16–1.40). Regarding inferences drawn from the estimated effect of pertuzumab on survival outcomes, false negative results are an uncontrolled risk since this retrospective study was not powered to detect differences between the two treatment groups.

## Discussion

This retrospective study evaluates the safety and efficacy of T-DM1 in a contemporary HER2-positive metastatic breast cancer group of patients. This is especially important to review as T-DM1 approval was based largely on a pertuzumab-naïve population, prior to adoption of the CLEOPATRA regimen as frontline therapy for HER2-positive metastatic disease. Our results indicate that T-DM1 remains an active and safe treatment option for patients previously treated with pertuzumab, as evidenced by a response rate, clinical benefit rate, survival distributions, and adverse reactions that were all comparable to estimates from pertuzumab-naïve patients. Although comparison of OS across the entire follow-up period did not reach statistical significance, we did observe higher 1-year and 2-year OS rates in the pertuzumab-pretreated cohort.

Our observed outcomes for patients who received pertuzumab prior to T-DM1 are consistent with most reports in the literature. Notably, our findings are similar to the overall response rate of 17.9% (95% CI: 9.4–26.4%) and median duration of T-DM1 of 4.0 months (95% CI: 2.7–5.1) in pertuzumab-pretreated patients as described by Dzimitrowicz et al. [11]. However, it’s important to note that the ORR to T-DM1 in pertuzumab-naïve populations, as studied in both the EMILIA (43.6%) and TH3RESA (31.3%) trials was higher than the response rate observed in our study (14.3%). This may be due to limited sample size in our cohort, but also to differences in the frequency and consistency of radiographic assessments in EMILIA and TH3RESA in the setting of a clinical trial, especially when considering variability between investigator assessed and blinded radiology review on trial.

T-DM1 activity after progression on a regimen of trastuzumab and pertuzumab is further supported by an Italian multi-center study which demonstrated prolonged duration of therapy, defined as T-DM1 > 6 months, in one-third of its patients [12]. However, a cohort of Japanese patients who received T-DM1 after progression on trastuzumab and pertuzumab had lower rates of response (11.1% vs. 25.0%) and shorter median PFS (2.8 months vs. 7.8 months) compared to a control group of pertuzumab-naïve patients [13].

Additionally, the median PFS of 9.5 months in our pertuzumab-pretreated cohort is comparable to the EMILIA trial (median PFS 9.6 months), which evaluated a similar population with a median of one prior therapy for metastatic disease, and slightly higher than the TH3RESA trial (median PFS 6.2 months), which evaluated a more heavily pretreated population compared to our cohort [9, 14]. An exploratory analysis of enrolled patients who were treated with T-DM1 after progression in CLEOPATRA and PHEREXA, two trials that assessed the benefit of adding pertuzumab to a regimen of trastuzumab and chemotherapy in the metastatic setting, provided further evidence that T-DM1 has clinical activity (median duration of therapy was 7.1 and 4.2 months in the respective trials) in patients with HER2-positive metastatic breast cancer after progression on dual HER2-directed therapy [15].

Our study also evaluated adverse events of special interest related to T-DM1 therapy, specifically, cardiac dysfunction and peripheral sensory neuropathy. In doing so, we have confirmed that T-DM1 is well tolerated as there were low rates of treatment discontinuation (4% in pertuzumab-pretreated, 10% in pertuzumab-naïve) due to drug-related toxicity and no grade ≥ 3 adverse events. Furthermore, there was no significant difference in the rates of these adverse events when comparing the two treatment groups.

However, important limitations to our study should be noted. First, the small number of patients in both cohorts reduced the statistical power to detect significant differences in both our primary and secondary clinical endpoints. Second, certain differences in the cohort characteristics, though not statistically significant, may have favored benefit in the pertuzumab pre-treated cohort. These include longer time from initial diagnosis to metastatic disease, as well as higher proportion of de-novo metastatic disease in the pertuzumab pre-treated cohort. It is important to note though that time from initial diagnosis to metastatic disease was more than 12 months in both cohorts, which would be consistent with the population in the CLEOPATRA trial [16]. Lastly, our retrospective study based on real-world data would not have followed similar schedules of assessment as large-scale registration trials do, which limits direct comparison with clinical trial results. Prospective cooperative group-led trials attempting to determine real-world safety and efficacy of T-DM1 therapy in patients with metastatic HER2-positive breast cancer are ongoing. One such effort is the EORTC 75111 trial (NCT01597414) evaluating treatment outcomes in elderly patients with metastatic HER2-positive breast cancer [17]. In this study, a group of 29 women who received T-DM1 as a pre-determined second-line treatment option had a median PFS of 5 months. Moreover, a Swiss trial (NCT01835236) is assessing sequential treatment with trastuzumab and pertuzumab with and without chemotherapy followed by T-DM1, with overall survival at 24 months as the primary endpoint.

As the MARIANNE trial did not show benefit of T-DM1 and pertuzumab compared to T-DM1 alone, the sequential approach of delivering pertuzumab-based therapy followed by T-DM1 represents the current clinical therapy algorithm for metastatic HER2-positive breast cancer [18]. Currently, treatment with trastuzumab, pertuzumab, and a taxane followed by T-DM1 at time of disease progression has been adopted as standard-of-care first-line and second-line therapy, respectively, in patients with HER2-positive metastatic breast cancer and is endorsed by national guidelines [19–21]. Patients who progress on T-DM1 can be eligible for recently approved novel agents (Trastuzumab-deruxtecan, Tucatinib) [22, 23].

## Conclusions

In summary, this study confirms previous reports of T-DM1 activity in patients with prior exposure to both trastuzumab and pertuzumab with a safety profile consistent with previous clinical trials. When compared to a control group of patients previously treated with trastuzumab-based therapy without prior exposure to pertuzumab, there were no significant differences in clinical activity or adverse events helping to solidify T-DM1 as a second-line therapy for patients with metastatic HER2-positive breast cancer in patients previously treated with dual anti-HER2 therapy.

## Data Availability

The datasets used and/or analyzed during the current study are available from the corresponding author on reasonable request.

## Abbreviations

CBR: Clinical benefit rate
ER: Estrogen receptor
HR: Hazard ratio
HER2: Human epidermal growth factor receptor 2
OS: Overall survival
ORR: Overall response rate
PFS: Progression-free survival
PR: Progesterone receptor
T-DM1: Ado-trastuzumab
